# Radioiodine treatment of differentiated thyroid cancer despite history of ‘iodine allergy’

**DOI:** 10.1530/EDM-13-0084

**Published:** 2014-03-01

**Authors:** Adam R Puchalski, Inder J Chopra

**Affiliations:** 1 Division of Endocrinology, Diabetes and HypertensionDavid Geffen School of Medicine at UCLA24-130 Warren Hall, 900 Veteran Avenue, Los Angeles, California, 90095USA

## Abstract

Radioiodine (^131^I) is a critical component in the treatment of differentiated thyroid cancer. We recently saw a patient with thyroid cancer who was hesitant to take ^131^I treatment because he had previously encountered an allergic reaction to administration of iodine-containing radiocontrast agent for computed tomography (CT) scanning. We were able to administer ^131^I treatment after discussion that his anaphylactic reaction was not due to iodine and that radioiodine (^131^I) treatment is unlikely to cause a reaction in the patient.

**Learning points:**

- An allergy to iodine itself does not exist.
- When patients claim that they have an ‘iodine allergy’, ask them what substances they are allergic to and what kind of reaction occurred during use of such substances.
- Radioactive iodine is not a contraindication for patients who claim an ‘allergy to iodine’.

## Background

Papillary thyroid carcinoma is the most common type of thyroid cancer, accounting for ∼80% of all malignant thyroid tumors. Total thyroidectomy followed by treatment with radioactive iodine is a key element of treatment of thyroid cancer. It is shunned in thyroid cancer patients with a history of allergy to iodinated compounds. We describe a patient who had papillary thyroid cancer and has a history of an anaphylactic reaction to computed tomography (CT) contrast agent. We considered his treatment with thyroid hormone replacement and normal salt-containing diet and advised radioactive iodine treatment, which did not carry any reaction in the patient. It makes us question whether radioiodine treatment is harmful for patients with a history not only of ‘iodine allergy’ but also of the existence of an allergy to iodine itself.

## Case presentation

A 55-year-old male with a past history of hypothyroidism and newly diagnosed papillary thyroid carcinoma with multiple lymph node metastases underwent total thyroidectomy and left neck dissection on 21st June 2012 at an outside hospital. He came to UCLA for further management of his papillary thyroid carcinoma.

The patient stated that he had anaphylactic shock 25 years ago after being administered iodinated contrast for a CT scan. He experienced shortness of breath, hives, loss of consciousness, and dry heaves. Since that time, he has not taken any further iodinated contrast agent and avoided taking drugs that he knew contained iodine. He has been taking Synthroid in the past, but switched to Armour Thyroid several years ago. He denies any adverse effects with taking thyroid supplementation or eating ‘iodized’ salt in his food.

The patient initially refused radioactive iodine treatment. Because of the experience he had with the CT contrast agent, he believed that he had an iodine allergy and as a result could not undergo radioactive iodine treatment. We counseled the patient for over an hour explaining that an allergy to elemental iodine does not exist. Additionally, we explained to the patient that both his thyroid supplementation and daily salt intake contain iodine and he did not have any reactions with these substances.

## Investigation

No laboratory tests were ordered at the time.

## Treatment

The patient agreed with this analysis and successfully underwent treatment with 100 mCi radioactive iodine ∼1 month following thyroidectomy.

## Outcome and follow-up

The patient was followed up for 6 weeks after radioactive iodine treatment. The patient reported no problems during or after the treatment.

## Discussion

The current literature argues that a pure allergic reaction to iodine does not exist. Simple atoms such as iodide itself or simple iodide salts are stated to not have the complexity required for antigenicity (2). Iodine may cause adverse reactions such as iododerma or iodide mumps. However, such reactions are usually related to large amounts of iodine and may not be allergic in nature (3).

Iodine is a trace element that is present in many items including seafood, salt, antiseptics, or radiocontrast materials. Allergies to seafood have been mistakenly blamed on the iodine in the seafood. Allergic reactions to seafood are apparently caused by IgE-mediated reactions against several proteins, such as parvalbumins in fish and tropomyosins in crustaceans and mollusks (3).

Several topical antiseptics contain iodine. In addition to that, they contain povidone, a polymer similar to the structure of dextran. When combined with iodine, the polymer carries iodine to bacteria, causing bacterial death. Povidone–iodine causes rare irritant dermatitis. There are several reports of non-iodinated povidones causing contact dermatitis and anaphylaxis. These reports have concluded that the allergy is likely against povidone and that iodine may not play a role (3). Van Ketel *et al*. (4) specifically studied this issue and compared allergic reactions to povidone–iodine and potassium iodide. In the study, there were eight patients who had allergic reactions to povidone–iodine. Of these patients, five were also tested with potassium iodide. The results indicated that ‘none of five patients with a history of contact dermatitis after povidone–iodine reacted adversely to patch testing with potassium iodide solution (4)’. Additionally, three of the eight patients were tested with iodine tincture and the results were ‘completely negative (4)’. The authors concluded that ‘allergy to povidone–iodine seems not to be based on sensitization to iodine (4)’.

There was a case report in 2007 where a patient was receiving radioactive iodine for a thyroid scan and for treatment of papillary thyroid carcinoma (5). Approximately, 30 min after ingestion of both radioactive ^123^I and ^131^I, the patient developed a severe urticarial rash, which disappeared after several hours. The key to this puzzle turned out to be that both treatments were administered in capsules that came from the same distributor. The capsules contained sucrose powder, titanium dioxide, FDC red 40, and DNC yellow 10. When the patient was given radioactive iodine in liquid form, she did not develop any complications, skin rashes, or other reactions after radioiodine treatment, implying that the contents of the capsules used in previous treatment may have been the agent inciting the allergic reaction noted (5).

The term ‘iodine allergy’ is a misleading term that confuses and worries patients and can cause controversy in the medical community. Iodine is an essential element in thyroid physiology and health. The literature that we explored argued that allergic reactions due to iodine-containing substances were caused not by iodine but rather by other ingredients contained in those substances and/or their vehicles.

Our patient clearly had an allergy to radiocontrast material used for CT scanning. Our analyses suggest that this information is not a contraindication to receiving radioactive iodine for scanning and/or treatment purposes. Radiopharmaceuticals do not have pharmacological effects. A typical dose of radioactive ^131^I used in treatment of papillary thyroid cancer is ∼50–200 mCi, averaging 100 mCi with the specific activity of ^131^I being about 5 Ci/mg. It can be calculated that 100 mCi of radioactive ^131^I would contain ∼20 μg of sodium iodide. This would mean that the dose of iodine in 100 mCi of radioactive iodide is ∼20 000 times smaller than that in a standard CT contrast dose containing about 350 mg of iodide (C Schiepers, personal communication). Even if there were an ‘iodine allergy’, such a tiny amount of stable iodine as contained in the radioactive iodine treatment is unlikely to elicit a significant reaction (C Schiepers, personal communication). Additionally, it can be calculated that the amount of stable iodine in an average daily production or replacement dose of thyroxine of 100 μg/day is 65 μg. Our patient had normal thyroid function for some 25 years before his thyroid gland was removed for treating thyroid cancer. Moreover, he was taking a normal salt diet without worrying about its iodine content. Normal daily salt intake is ∼5–10 g/day. Assuming an iodine supplementation of some ten parts per million, this amount of salt would be expected to contain 50–100 μg of sodium iodide that our patient consumed daily without any reaction.

Unfortunately, there is a lot of misinformation and confusion about the issue of iodine allergy not just in the public, but also in the medical community. When faced with this issue, physicians do not perform useful procedures with radioiodine and/or patients may be hesitant to accept treatment that is otherwise needed. In treating papillary thyroid carcinoma, radioactive iodine is a key and necessary component of treatment. Careful attention/consideration to all aspects of treatment must be undertaken before discarding radioactive iodine from its use in treatment of these patients. Patients with ‘iodine allergy’ do not have an allergy to iodine itself but to its accompanying materials.

## Patient consent

Patient is deceased.

## Author contribution statement

Both Drs I J Chopra and A R Puchalski were involved in the clinical care of the reported case.
